# Prefrontal cortex activation under stress as a function of borderline personality disorder in female adolescents engaging in non-suicidal self-injury

**DOI:** 10.1192/bjo.2024.728

**Published:** 2024-08-08

**Authors:** Saskia Höper, Felix Kröller, Anna-Lena Heinze, Kay Franziska Bardtke, Michael Kaess, Julian Koenig

**Affiliations:** Department of Child and Adolescent Psychiatry, Centre for Psychosocial Medicine, Heidelberg University, Germany; Department of Child and Adolescent Psychiatry, Centre for Psychosocial Medicine, Heidelberg University, Germany; and University Hospital of Child and Adolescent Psychiatry and Psychotherapy, University of Bern, Switzerland; University Hospital of Child and Adolescent Psychiatry and Psychotherapy, University of Bern, Switzerland; Faculty of Medicine, University of Cologne, Germany; and Department of Child and Adolescent Psychiatry, Psychosomatics and Psychotherapy, University Hospital Cologne, Germany

**Keywords:** Stress, non-suicidal self-injury, borderline personality disorder, functional near-infrared spectroscopy, prefrontal cortex

## Abstract

**Background:**

Neuroimaging studies suggest alterations in prefrontal cortex (PFC) activity in healthy adults under stress. Adolescents with non-suicidal self-injury (NSSI) report difficulties in stress and emotion regulation, which may be dependent on their level of borderline personality disorder (BPD).

**Aims:**

The aim was to examine alterations in the PFC in adolescents with NSSI during stress.

**Method:**

Adolescents (13–17 years) engaging in non-suicidal self-injury (*n* = 30) and matched healthy controls (*n* = 29) performed a task with low cognitive demand and the Trier Social Stress Test (TSST). Mean PFC oxygenation across the PFC was measured with an eight-channel near-infrared spectroscopy system. Alongside self-reports on affect, dissociation and stress, BPD pathology was assessed via clinical interviews.

**Results:**

Mixed linear-effect models revealed a significant effect of time on PFC oxygenation and a significant time×group interaction, indicating increased PFC activity in patients engaging in NSSI at the beginning of the TSST compared with healthy controls. Greater BPD symptoms overall were associated with an increase in PFC oxygenation during stress. In exploratory analyses, mixed models addressing changes in PFC connectivity over time as a function of BPD symptoms were significant only for the left PFC.

**Conclusions:**

Results indicate differences in the neural stress response in adolescents with NSSI in line with classic neuroimaging findings in adults with BPD. The link between PFC oxygenation and measures of BPD symptoms emphasises the need to further investigate adolescent risk-taking and self-harm across the spectrum of BPD, and maybe overall personality pathology, and could aid in the development of tailored therapeutic interventions.

Non-suicidal self-injury (NSSI) is defined as the intentional, self-directed damage of one's own body tissue without suicidal intent and for purposes not culturally or socially sanctioned.^1^ Given that NSSI is commonly associated with high psychological strain and a large range of comorbid mental disorders,^2^ it is regarded as a transdiagnostic marker of risk, particularly for the presence of disorders that are related to severe emotion dysregulation, such as depression or borderline personality disorder (BPD). The diagnosis of BPD is widely accepted as reliable and valid for adolescents,^3^ with prevalence rates ranging from 0.9 to 3.2% among adolescents and young adults in the USA.^4^ Compared with other patient groups, patients with BPD commonly report high levels of psychopathological distress and lower health-related quality of life and psychosocial functioning.^5,6^ BPD is associated with altered emotion regulation and increased stress vulnerability.^7,8^ Research suggests altered stress reactivity in patients with NSSI and/or BPD. Adult patients with BPD often report a higher stress burden and greater emotional reactivity to daily stressors compared with healthy controls and compared with patients with psychotic disorders.^9^ Altered stress reactivity in adult patients with BPD is characterised by increased negative emotions after stress,^10^ as well as an attenuated cortisol response.^11^ The stress-reducing effect of self-injury in adult BPD is described by a decrease in amygdala activation after self-injury.^12^

## Neuroimaging in non-suicidal self-injury

Former classical neuroimaging studies in NSSI showed task-specific alterations of neural activity in patients compared with controls. During a social exclusion paradigm, young patients engaging in NSSI showed increased prefrontal cortex (PFC) activation^13^ as well as an aberrant activation in the amygdala at rest and during emotion recognition.^14^ Research on the neurobiological stress response in adolescent patients with BPD is sparse. For example, adolescent patients with BPD showed an attenuated heart rate response during a dual-task under stress.^15^ In addition, an attenuated cortisol response to stress was also found in adolescents with NSSI.^16^ Concerning neuroimaging, findings in healthy samples from functional magnetic resonance imaging (fMRI), electroencephalography and functional near-infrared spectroscopy (fNIRS) studies suggest alterations in prefrontal brain activation in response to stress. In healthy participants, stress affects the dorsolateral and ventral right PFC,^17–19^ and the orbitofrontal cortex.^17,18^ To our knowledge, there are no existing studies examining neural responses to acute stress in adolescents with NSSI or BPD in a real-world setting. Stress paradigms that are suitable to be conducted in the MRI (such as the Montreal Imaging Stress Test) suggest alterations in functional connectivity in adolescent patients engaging in NSSI compared with controls.^20^

Because of its narrowness, in-scanner stress tasks pose considerable challenges to the research methodology. Some well-established stress paradigms cannot be performed in the scanner without compromising their validity. Other paradigms need extensive modification to be scanner-compatible. fNIRS represents a valuable alternative neuroimaging method to study neural responses during stress induction. fNIRS is highly correlated with blood-oxygenation-level dependent signals from fMRI, when focusing on the cortical surface.^21^ The main principle of fNIRS is the measurement of light in the near-infrared spectrum. Through light attenuation from absorption and scattering, it detects changes in the concentration of oxygenated haemoglobin (O_2_Hb) and deoxygenated haemoglobin (HbR) in surface areas of the brain in real time.^22^

## The present study

Most research on participants engaging in NSSI focuses on adult patients with BPD. To account for developmental aspects and the interlink between NSSI and BPD in adolescents, studies on NSSI across the spectrum of BPD pathology in adolescent samples are needed. In the present study, we aimed to investigate the neural responses of adolescents with NSSI to an acute stressor, comparing patients with a healthy control group and investigating the stress response as a function of BPD severity. We hypothesised that adolescent patients engaging in NSSI would show an increased PFC activation in response to an acute stress task compared with matched healthy controls. We further expected that the severity of BPD pathology would be positively correlated with the increase in PFC activation in the NSSI group. In a former study, we found no group differences in connectivity across the PFC during resting state between patients engaging in NSSI and healthy controls, but found a descriptively stronger connectivity that was associated with greater BPD pathology.^23^ Hence in exploratory analyses, we aimed to examine PFC connectivity under stress and in association with BPD pathology.

## Method

### Participants

Patients were recruited from the specialised out-patient clinic for risk-taking and self-harming behaviour (AtR!Sk; *Ambulanz für Risikoverhaltensweisen und Selbstschädigung*^24^ at the Clinic for Child and Adolescent Psychiatry, Center for Psychosocial Medicine, University of Heidelberg, Germany). Healthy controls were recruited via public advertisement (e.g. recruitment at public places) and matched to the patient group by age. As self-harming behaviours differ between genders,^25^ only females were included to this analysis. Previous data from the study, focusing on behavioural and psychophysiological outcomes, were published earlier.^26^ The recruitment period lasted from July 2016 until May 2018. Inclusion criteria for the patient group were at least five incidents of NSSI during the past 12 months, age between 13 and 17 years, and female gender. Inclusion criteria for the healthy control group were age between 13 and 17 years and female gender. General exclusion criteria were deficient language skills or clinically relevant impairments in intelligence, glucocorticoid medication intake, pregnancy, any underlying neurological or endocrinological diseases, acute psychosis, acute suicidality, substance dependency and a body mass index (BMI) <17.5 kg/m^2^ or >30 kg/m^2^. An additional exclusion criterion for the patient group was current BPD drug treatment. A further exclusion criterion for participants of the control group was any current and former psychiatric disorder or lifetime NSSI. After completion of the study, every participant received an allowance of €40 for study participation.

For the NSSI group, 180 (100%) adolescents were screened and 37 (20.56%) were included in the study. Reasons for study exclusion are provided (see Supplementary Table 1 available at https://doi.org/10.1192/bjo.2024.728). During study participation, seven participants in the NSSI group dropped out of the study: one (0.56%) reported acute headaches, two (1.11%) showed dissociative symptoms that required interruption of experimental procedures, three (1.67%) did not show up for the second appointment and one (0.56%) dropped out because of further reasons not documented in detail. This resulted in a final sample of *n* = 30 (16.67%) for the NSSI group. A total of 66 (100%) female adolescents were screened as healthy controls for study participation. Of those, 31 (46.97%) were included in the study. One (1.52%) refused to participate in the stress task and hence dropped out of the study. Finally, one (1.52%) had to be excluded from NIRS analyses because of technical issues during the assessment. This resulted in a sample of *n* = 29 healthy controls (43.94%).

The study comprised two appointments. The first appointment included an extensive clinical characterisation of participants via interviews and self-reports. Appointment two comprised the actual stress induction experiment. Written informed consent was provided by participants and their caregivers before inclusion in the study. The authors assert that all procedures contributing to this work comply with the ethical standards of the relevant national and institutional committees on human experimentation and with the Helsinki Declaration of 1975, as revised in 2008. All procedures involving human patients were approved by the ethical committee of the University of Heidelberg (study identifier: S-685/2015).

### Procedures

At the first appointment, patients completed a diagnostic interview and answered self-report questionnaires. Diagnostic tools that are relevant for the present manuscript are presented here. Further instruments are reported elsewhere.^26^ For all diagnostic tools, a validated German translation was used. During the interview, demographic data (e.g. school form, age, height, weight) were queried. Suicidality and NSSI behaviour were assessed with the Self-Injurious Thoughts and Behaviors Interview (SITBI-G),^27^ BPD criteria were assessed with the Structured Clinical Interview for the DSM-IV (SCID-II).^28^ The semi-structured Mini-International Neuropsychiatric Interview for children and adolescents (M.I.N.I.-Kid)^29^ was conducted with all participants to check for common Axis 1 disorders. In self-report questionnaires, dimensional characteristics of BPD criteria were determined with the Borderline Symptom List Short Form (BSL-23).^30^ Moreover, traumatic childhood experiences were determined with the Childhood Trauma Questionnaire (CTQ).^31^ In addition to the diagnostic interview, possible influencing factors of the physiological stress reaction were assessed. Therefore, somatisation, depression and anxiety were assessed with the Brief Symptom Inventory (BSI-18).^32^ Healthy controls also underwent a diagnostic interview and questionnaires, which included a demographic assessment, the SITBI-G, BSL-23, BSI-18 and CTQ. To make sure that healthy control participants did not fulfil criteria for any psychiatric disorder, the SCID-II Non-Patient (SCID-N/P)^33^ was conducted. All interviews and questionnaires were digitised with LimeSurvey for Windows (LimeSurvey GmbH, Hamburg, Germany; www.limesurvey.org/).

The second appointment comprised the actual experiment, including the stress induction paradigm. The detailed procedures are described elsewhere.^26^ The Trier Social Stress Test (TSST) for stress induction in children and adolescents was used as the stress task.^34^ The rationale for using the TSST over other paradigms is that the TSST is one of the best validated stress paradigms to reliably induce interpersonal stress in adolescents.^35^ The aim was not to specifically induce academic stress. The respective cover-story has been suggested for adolescents and has previously been widely used. As adolescents with NSSI or BPD are known to show difficulties in handling interpersonal stress, examining stress during real-world interpersonal interaction is important in this patient group. Thus, it was intended to present the participants with a real-world scenario in a controlled stress-provoking setting. Before the TSST, participants performed the Color Detection Task (CDT) as a cognitive low-demand baseline task.^36^ They were asked to count the occurrence of a specific colour of a rectangle over 5 min. The TSST consisted of a preparation phase and two stress tasks. During the preparation phase, participants had 5 min to prepare themselves for an interview for their dream school. During phase 1 of the TSST, they were asked to present themselves in front of two interviewers. They were asked to elaborate on why they were the perfect candidate for a new school. Participants believed that their speech was videotaped. The interviewers did not show any reaction to the participants' speech and did not answer any questions. After 5 min they interrupted the participant and continued with a mental arithmetic test during which the participant had to subtract mentally. Every time the participant made a mistake, they had to start subtracting from the beginning. Before and after the stress task, the participants answered two short questionnaires regarding their momentary mental state (Positive and Negative Affect Schedule (PANAS);^37^ Dissociation-Tension Scale (DSS-4)^38^), and rated their stress levels on a visual analogue scale ranging from 0 to 100. At the end, all participants were debriefed.

### fNIRS measurement

**Fig. 1:**
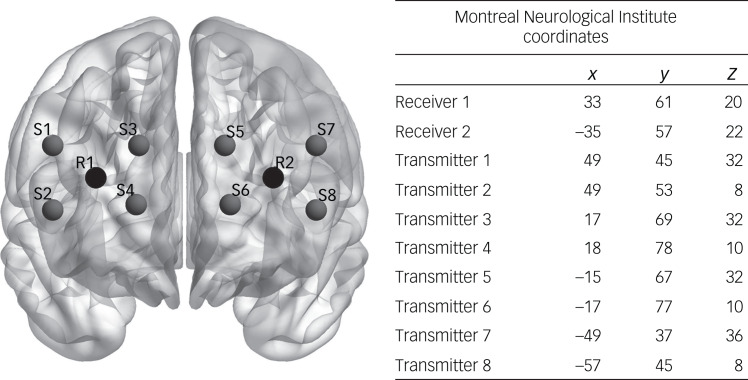
Optode placement on the forehead.^23^ R indicates receiver and S indicates source.

An eight-channel continuous-wave fNIRS system was used for continuous recordings of PFC oxygenation (OctaMon, Artinis Medical Systems, The Netherlands). It is an optical neuroimaging device that contains eight LED light sources and two PIN diode receivers that are placed with a headband onto the forehead of the participant. The light sources emit light in the near-infrared spectrum, which passes the skull cap and is absorbed by the O_2_Hb and the HbR. The attenuation of light by different tissues is described by the modified Beer-Lambert Law. As O_2_Hb absorbs light with a wavelength >800 nm and HbR absorbs light with a wavelength <800 nm, the OctaMon device emits light in two wavelengths, 760 nm and 850 nm. Receivers and transmitters are summed up as optodes. The inter-optode distance is 35 mm, which results in an estimated cortical penetration depth of 17 mm. The optodes were placed onto the forehead according to the international 10–20 system for electroencephalography electrodes placement.^39^ The optode configuration and their estimated coordinates according to the Montreal Neurological Institute brain template are displayed in Fig. 1. When placing the headband to the forehead, the instructor made sure that there was no hair between optodes and skin, and that the received light was between 3 and 97% of the emitted light. Values close to 0% indicate that almost no light reaches the receiver, whereas values close to 100% indicate that environmental light is also received. According to the general equation for the differential path length factor and in regard to current study protocols, the differential path length factor was set to 6 cm.^40^ The sampling rate was set to 50 Hz per channel.

### fNIRS data preprocessing

Raw haemoglobin density values (O_2_Hb and HbR) were recorded by the fNIRS device and sent to a laptop via Bluetooth, where it was stored as *.oxy3-data using the Oxysoft software, version 3.1.103 for Windows (ArtinisMedical Systems, Elst, The Netherlands).^41^ All fNIRS data were segmented according to the start and end times of the different blocks during the study. In preparation for the preprocessing of the data, they were imported to MATLAB^42^ for Windows (The Math Works Inc., Natick, Massachusetts, USA) with the *oxysoft2matlab* function provided by Artinis Medical Systems (The Netherlands). Preprocessing was conducted with the HOMER2 toolbox.^43^ First, the raw optical density measures were converted to optical density values (*hmrIntensity2OD*). Next, we corrected for motion artefacts as recommended.^44^ We applied a two-step correction process. The first step was the application of a wavelet-based motion correction with a probability threshold of *α* = 0.01 (*hmrMotionCorrectWavelet*). In the second step, we corrected for motion artifacts by using the *hmrMotionArtifact* function. This function removes signal changes where the standard deviation exceeds the factor 20 (*SDThresh*) or where the peak-to-peak amplitude exceeded 0.5 (*AMPThresh*) within 0.5 s (*tMotion*). High-frequency noise was removed with a third-order Butterworth filter (*LowFrequencyPass*), which only allowed frequencies lower than 0.5 Hz so that high-frequency physiological noise (e.g. cardiac) was removed. Finally, optical density rates were converted to haemoglobin concentrations in μmol/L (10^−6^ mol/L) for O_2_Hb, HbR and total haemoglobin (O_2_Hb + HbR), and were exported for segmentation and further analyses to Stata/SE software, version 16.0 for Windows (StataCorp LLC, College Station, Texas, USA).^45^ After preparing the fNIRS data for analysis, recordings from channel five of one healthy control were removed from the analysis during the CDT and the preparation of the TSST, as the recorded values were unrealistically high, suggesting that external light was absorbed. Visualisations were prepared with BrainNet Viewer, version 1.6 for Windows (www.nitrc.org/projects/bnv/).^46^

### Statistical analysis

All statistical analyses were conducted in Stata/SE software version 16.0.^45^ In line with previous research,^21,47–51^ we calculated mean values and standard deviations for O_2_Hb for all relevant time blocks (four time blocks: CDT; Preparation TSST; TSST, divided into the free speech and the mental arithmetic test). For O_2_Hb, mean values per channel and a grand mean value per block were calculated. First, to check for differences between groups on demographical data and on the clinical interviews, *t*-tests (continuous variables) and *χ*^2^-tests (categorical variables) were calculated. Second, a linear mixed-effects model was calculated for O_2_Hb to test the hypothesis that adolescent patients engaging in NSSI would show an increased PFC activation in response to an acute stress task. Predictors were time (CDT, preparation TSST, free speech, arithmetic task) and group (patients versus healthy controls), as well as their interaction included as fixed effects. The participants’ identifiers were addressed as random effect. Where applicable, planned contrasts were used to investigate effects of group at single events of time, to address when effects emerged and how long they lasted. To account for the severity of BPD symptoms and to test the hypothesis that severity of BPD pathology would be positively correlated with the increase in PFC activation in the NSSI group, models were repeated using a dimensional approach of BPD severity based on BSL-23 and the number of BPD criteria (SCID-II). The interaction of time × severity was addressed. Consistency between the two measurement modalities (BSL-23 and SCID-II) was inspected. For consistent interactions between the modalities, margin plots at fixed levels of BPD symptoms (BSL-23: 0, 1, 2, 3, 4; BPD criteria: 0, 3, 6, 9) were composed. Next, effects of self-reported levels of dissociation, stress, and positive as well as negative affect on PFC oxygenation were addressed. Each measure was included separately as a predictor to the mixed linear-effects model on O_2_Hb, and it was checked whether the inclusion of self-reports improved the overall model fit. Group differences on self-reports in response to stress are reported elsewhere.^26^ Finally, connectivity between fNIRS channels across the PFC was analysed; cross correlation coefficients were determined for each time block and analysed in mixed-effects models with time and group as predictors. Contrast analyses were used to address changes in connectivity strength by time and group. Furthermore, BPD dimensionality (BSL-23 and SCID-II) was also included in additional mixed-linear effects models as indicated above. Because of technical issues, one healthy control had to be excluded from the connectivity analysis. Hence, the sample resulted in *n* = 30 patients engaging in NSSI and *n* = 28 healthy control participants. For simplicity and in line with previous research, only O_2_Hb was included in all analyses, as it is the most informative haemoglobin variable and changes in haemoglobin are easier to detect.^23,52^

## Results

### Sample characteristics

For a detailed description of sociodemographic and clinical characteristics of the sample, see Table 1. As illustrated in Table 1, groups differed on BMI as well as on measures of psychopathological distress.

**Table 1:** Sociodemographic and clinical characteristics by group

	NSSI group	Healthy control group	*P*-value
*n* (% female)	30 (100.00)	29 (100.00)	
Age, years, mean (s.d.)	15.733 (0.19)	15.759 (0.24)	0.934
School, *n* (%)			0.236
Hauptschule	1 (3.33)	0 (0)	
Realschule	7 (23.33)	5 (17.86)	
Gymnasium	20 (66.67)	22 (78.57)	
Other	2 (6.67)	2 (7.14)	
BMI, mean (s.d.)	22.127 (0.62)	19.802 (0.35)	0.002
BSI-GSI	1.535	0.243	<0.0001
BSL-23	1.826	0.142	<0.0001
BSL overall personal state	38.33 (1.64)	80.00 (1.25)	<0.0001
CTQ	43.87 (1.36)	28.28 (0.40)	<0.0001
BPD, full diagnosis	7 (23.33)	0 (0.00)	0.004
BPD fulfilled criteria, *n* (%)			
Frantic efforts to avoid abandonment	4 (13.33)	0 (0.00)	<0.0001
Pattern of unstable/intense relationships	16 (53.33)	0 (0.00)	<0.0001
Identity disturbance	9 (30.00)	0 (0.00)	<0.0001
Impulsivity	6 (20.00)	0 (0.00)	<0.0001
Recurrent suicidal/self-injurious behaviour	28 (93.33)	0 (0.00)	<0.0001
Affective instability	21 (70.00)	0 (0.00)	<0.0001
Chronic feelings of emptiness	10 (33.33)	0 (0.00)	<0.0001
Inappropriate, intense anger	9 (30.00)	0 (0.00)	<0.0001
Paranoid ideation/severe dissociative symptoms	9 (30.00)	0 (0.00)	<0.0001
Comorbidity (ICD-10), *n* (%)			
F0X	0 (0.00)	0 (0.00)	
F1X	3 (10.00)	0 (0.00)	<0.0001
F2X	0 (0.00)	0 (0.00)	
F3X	17 (56.67)	0 (0.00)	<0.0001
F4X	14 (46.67)	0 (0.00)	<0.0001
F5X	1 (3.33)	0 (0.00)	0.321
F6X	6 (20.00)	0 (0.00)	0.011
F7X	0 (0.00)	0 (0.00)	
F8X	1 (3.33)	0 (0.00)	0.321
F9X	3 (10.00)	0 (0.00)	0.080
Number of suicide attempts, mean (s.d.)			
Lifetime	2.545 (1.92)	0 (0.00)	
Past 12 months	1.200 (1.61)	0 (0.00)	
Past 6 months	0.545 (0.93)	0 (0.00)	
Number of NSSI events, mean (s.d.)			
Past 12 months	89.033 (144.90)	0 (0.00)	
Past 6 months	39.720 (49.79)	0 (0.00)	

All values are means with s.d. in brackets, if not otherwise classified. School: after 4 years of primary school, secondary school is divided into three school forms in Germany; Hauptschule consists of 5 years of secondary school, qualifying for further vocational education; Realschule consists of 6 years and ends with a general certificate of secondary education; Gymnasium takes 8–9 years and ends with a general university entrance qualification. NSSI, adolescents with five or more events of non-suicidal self-injury; BMI, body mass index; BSI-GSI, Brief Symptom Inventory – Global Severity Index; BSL-23, Borderline Symptom List Short Form; CTQ, Childhood Trauma Questionnaire; BPD, borderline personality disorder.

On average, participants of the NSSI group fulfilled 3.53 (s.d. = 1.85) BPD criteria. Predominantly, they reported the criterion of NSSI and/or suicidality (*n* = 28; 93.33%), followed by emotional instability (*n* = 21; 70.00%) and unstable and intense relationships (*n* = 16; 53.33%). The mean age of NSSI onset was 12 years (s.d. = 0.436).

### PFC activation

**Fig. 2:**
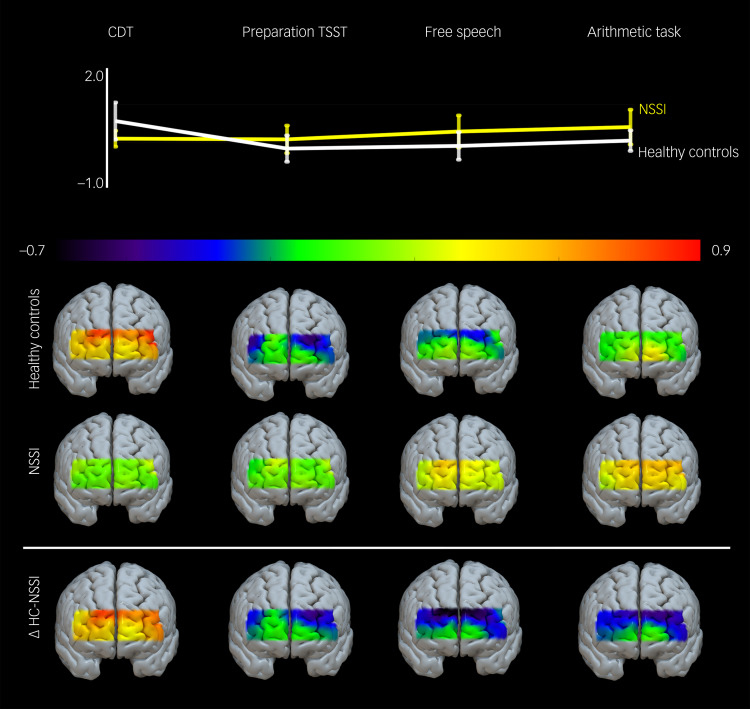
Prefrontal oxygenation over the course of time. Displayed is the prefrontal oxygenation in μmol/L over the course of the Color Detection Task (CDT) and the Trier Social Stress Test (TSST), divided into preparation phase, free speech task and arithmetic task. NSSI, adolescents engaging in non-suicidal self-injury; ΔHC-NSSI, difference between healthy controls and NSSI group.

General PFC activation over time is visualised in Fig. 2. The model on O_2_Hb was significant (Wald *χ^2^*(7) = 23.63; *P* = 0.001). Main effects were found for time (*χ^2^*(3) = 9.34; *P* = 0.025), but not for group (*χ^2^*(1) = 0.43; *P* = 0.512). The interaction between time and group was significant (*χ^2^*(3) = 14.33; *P* = 0.003). Adjacent contrast for time revealed a significant contrast between CDT and preparation of the TSST (*χ^2^*(1) = 8.30; *P* = 0.004), but not for time blocks later on (preparation TSST versus free speech: *χ^2^*(1) = 1.18, *P* = 0.277; free speech versus arithmetic task: *χ^2^*(1) = 1.00, *P* = 0.317). Although the O_2_Hb in the NSSI group remained unchanged from baseline (CDT) to the preparation phase of the TSST, and increased slowly during the course of the TSST, the level of O_2_Hb in healthy controls decreased between CDT and preparation phase, and slowly increased again during the free speech and the arithmetic task (see Fig. 2). When comparing O_2_Hb between groups during the CDT, baseline O_2_Hb in the NSSI group was descriptively lower compared with healthy controls. The difference just failed to reach statistical significance (*t* = −1.839; *P* = 0.071). Results of the exploratory connectivity analyses are provided in the Supplementary Material.

### BPD symptoms and changes in PFC activation

**Fig. 3:**
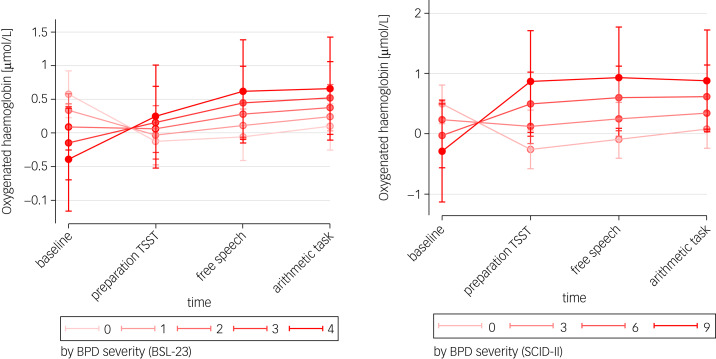
Margins plots between borderline personality disorder symptoms and prefrontal oxygenated haemoglobin over time. BPD, borderline personality disorder; BSL-23, Borderline Symptom List Short Form; SCID-II, Structured Clinical Interview for the DSM-IV; TSST, Trier Social Stress Test.

Both continuous models on BPD severity showed significant model fit for O_2_Hb (BSL-23: Wald *χ^2^*(7) = 23.05, *P* = 0.002; BPD criteria: Wald *χ^2^*(7) = 28.08, *P* < 0.001). There was a significant time × severity interaction for BPD severity in predicting changes in O_2_Hb for BSL-23 (*χ^2^*(3) = 14.03; *P* = 0.003), as well as for the number of BPD criteria (*χ^2^*(3) = 17.25; *P* = 0.001). The respective findings are illustrated in Fig. 3. As illustrated, higher self-reported BPD pathology (BSL-23) and greater number of BPD criteria (SCID-II) were associated with lower O_2_Hb during baseline and higher O_2_Hb during the preparation of the TSST and during the TSST itself.

### Self-reported dissociation, stress and mood and changes in PFC activation

To investigate the influence of self-reports (i.e. dissociation, stress, and negative and positive affect), each measure was included separately as predictor to the linear-mixed effects model on O_2_Hb. Adding self-reported dissociation as a predictor did not improve the respective model fit (Wald *χ^2^*(11) = 23.15; *P* = 0.017). Furthermore, no significant main effect of dissociation on the course of O_2_Hb occurred (coefficient, 0.022; *P* = 0.836). In the same vein, self-perceived stress as predictor on O_2_Hb did not improve the model fit (Wald *χ^2^*(11) = 28.42; *P* = 0.003), and no significant main effect of stress on the course of O_2_Hb was found (coefficient, −0.003; *P* = 0.655). For the inclusion of negative affect as a predictor to the model on O_2_Hb, the model fit remained significant, but did not improve (Wald *χ^2^*(11) = 27.31; *P* = 0.004). The main effect of negative affect was not significant (coefficient, 0.004; *P* = 0.948). Finally, positive affect was included as a predictor to the linear mixed-effects model on O_2_Hb. Again, the model fit did not improve (Wald *χ^2^*(11) = 37.13; *P* = 0.0001), and the main effect for positive affect on the course of O_2_Hb was not significant (coefficient, 0.051; *P* = 0.180).

## Discussion

To our knowledge, this is the first study investigating neural response indicated by PFC oxygenation to stress in adolescents engaging in NSSI across the spectrum of BPD pathology. We examined PFC activation before and during the TSST. Although PFC oxygenation during baseline was descriptively lower in patients engaging in NSSI, it slightly increased in response to stress. In contrast, PFC oxygenation in healthy controls decreased under stress. This result partially supports the hypothesis that there is greater PFC oxygenation in response to stress among adolescents with NSSI. We found evidence for such a group difference; however, it resulted mainly from a decrease in PFC activation in healthy controls. Interestingly, the anticipation of the stressor (preparation of the TSST) was sufficient to cause the decrease in healthy controls. In a former study, we found a significantly attenuated PFC oxygenation at rest in adolescents with NSSI compared with a healthy control group.^23^ Here, in a much smaller sample, the difference at baseline just missed statistical significance, but replicated the finding of lower resting PFC oxygenation in patients engaging in NSSI, in principle.

We can speculate that structural differences, such as lower grey matter volume in PFC areas, might cause lower PFC oxygenation during resting state in patients engaging in NSSI. Decreased grey matter volume in the anterior cingulate cortex of older adults has been related to lower blood flow during resting state.^53^ Research on patients with BPD and NSSI show volume losses in PFC areas,^54^ and decreased activation in the dorsolateral PFC.^55^ Hence, potential volume losses occurring in association with BPD and/or NSSI pathology might account for PFC hypoactivation during rest in patients engaging in NSSI. It has been shown that reduced prefrontal grey matter volume in older adults is associated with a greater increase in PFC activation during dual-task walking compared with single-task walking.^56^ In addition, lesion studies found altered stress response in people with lesions in the medial PFC.^57^ This line of reasoning links reduced PFC activation during rest, and overcompensation during demand (e.g. task-based activity), with structural deficits in patients with BPD and NSSI that might compromise their capacity to adequately adapt to psychosocial stress.

We also hypothesised that greater BPD pathology would be related to greater PFC oxygenation in the NSSI group. Here, we found that greater BPD pathology (self-report and clinical interview) was associated with decreased PFC oxygenation at baseline and increased PFC oxygenation during the task on a dimensional level. Hence, our hypothesis was supported. In adults with BPD, a negative correlation between grey matter volume loss in the PFC and temporal areas and self-reported BPD symptoms was found at rest, using MRI.^58^ Together with the results from the present study, these findings support the assumption of linear alterations of neurobiological systems as a function of BPD severity. As BPD-specific changes in PFC oxygenation seem to be prevalent even in adolescents with NSSI, the need for early detection and therapeutic interventions is emphasised. There are only limited therapeutic options specifically focusing on NSSI. The current work highlights differences in neural activity under stress as a function of BPD severity in patients engaging in NSSI, and therefore emphasises the use of tailored interventions for stress regulation in these patients. Furthermore, PFC oxygenation might serve as biomarker in clinical care to address the efficacy of respective interventions to improve stress response on a neurobiological level. Hence, future studies should examine the use of fNIRS to monitor PFC activation as a biomarker for severity, monitoring and therapeutic outcome in adolescent patients engaging in NSSI.

In exploratory analyses, connectivity across the PFC over time was investigated using mixed linear-effects models. Results revealed significant model fit mainly concerning connectivity between channels covering the left PFC when accounting for BPD symptom severity over time. The left PFC seems to play a crucial role in stress compensation, especially for those reporting greater BPD pathology. Prior research indicates a higher vulnerability of the left PFC to higher cortisol levels and chronic stress compared with the right hemisphere.^59^ Studies on frontal asymmetry found that left individual frontal activity predicted greater cortisol increases during stress.^60^ Decreased activity in the orbitofrontal PFC seems to be related to an increased cortisol secretion after stress.^17^ Interestingly, cortisol response is reported to be attenuated after stress in adolescent NSSI.^61^

As reported elsewhere,^26^ cortisol secretion of the present sample increased after the stress task in both groups. However, compared with controls, the cortisol increase after stress in patients engaging in NSSI was attenuated and greater BPD pathology (self-report and clinical interview) was associated with a more attenuated increase in cortisol secretion after stress.^26^ These results on the cortisol response are in line with prior findings and add to the overall picture. In the control group, prefrontal oxygenation decreased under stress and cortisol secretion increased, in line with findings from studies in healthy adults.^62^ This finding suggests a maladaptive interplay between the PFC and the hypothalamus-pituitary-adrenal axis in NSSI. Findings from this study extended by the results from Koenig et al^26^ suggest that neural mechanisms as a function of BPD severity are associated with the observed attenuation in physiological stress reactivity (i.e. blunted cortisol response) in NSSI. This blunted cortisol response in adolescents with NSSI has been described previously and replicated in independent studies,^16^ and might present a physiological marker for difficulties in emotion regulation in NSSI. Here, we provide evidence on a potential neural mechanism associated with this phenomenon, related to alterations in PFC oxygenation and its connectivity. Importantly, this pattern seems to manifest on a continuum as a function of BPD severity. Prior research found that greater connectivity between prefrontal and limbic areas in healthy adolescents during rest was associated with greater cortisol reactivity to an acute stressor, whereas this relationship was only weak or inversed in depressed adolescents with and without NSSI.^63^ In a sample of patients at ultra-high risk for psychosis, reduced grey matter volume in the PFC was associated with a blunted response of the hypothalamus-pituitary-adrenal axis.^64^ Taken together, structural deficits of the PFC resulting in altered activation patterns in adolescent NSSI may compromise the capacity to regulate emotions and stress, both on a psychological and physiological level, and seem to be related to BPD severity. Future research should assess real-time prefrontal reactivity to stress in relation to physiological reactivity in NSSI, to disentangle the temporal associations between cortex activation and physiological response – also in consideration of neural feedback loops.

The use of the TSST to examine alterations in neural responding in adolescent NSSI is a novelty of the present study. Former research on neural alterations in adolescent NSSI is sparse and mainly limited to social exclusion paradigms (such as the Cyberball task) or other stress-inducing paradigms (such as stress induced by time pressure, using the Montreal Imaging Stress Test), using fMRI. Although social exclusion marks an important stressor in BPD, the examination of neural responses to different stress tasks in a real-world setting may help to better understand differences in neural activity in adolescent NSSI, and ease translation of research findings to real-world clinical care.

This study comes with several limitations. First, only female adolescents were investigated. For neural activation, gender differences are often reported.^65,66^ Hence, findings from this study are not readily transferable to male adolescents with NSSI, and further research is needed. Second, sample size was a limiting factor when investigating the influence of BPD pathology. Finally, connectivity measures implemented in the present study are limited because of the limited spatial resolution of NIRS measurement compared with other neuroimaging techniques. Other neuroimaging modalities such as fMRI have a greater spatial resolution, and even provide insight into the connectivity with deeper subcortical brain structures such as the limbic system, which is known to be relevant in emotion regulation and BPD.

Taken together, this study shows differences in the neural response of the PFC to a psychosocial stress task comparing adolescents with and without NSSI. Although PFC oxygenation decreased in healthy controls, PFC oxygenation slightly increased in adolescents with NSSI. The trajectory of PFC oxygenation from rest to stress overall was associated with the severity of BPD pathology. To our knowledge, this is the first study to investigate PFC oxygenation during stress in a sample of adolescents with and without NSSI across the spectrum of BPD pathology. Further research is needed to replicate and extend these findings. Future studies should build on these findings, using different stress paradigms and neuroimaging modalities. The finding that the severity of BPD pathology is associated with alterations in PFC activation during stress emphasises that not only patients with full-blown BPD might benefit from tailored therapeutic interventions to improve stress regulation, but also patients engaging in NSSI who only partially fulfil BPD criteria. The consideration of BPD severity in tailoring interventions might be beneficial: PFC activation may serve as biomarker in this regard. Hence, future studies should examine the use of fNIRS to monitor changes in PFC oxygenation during treatment. Furthermore, future studies may extend the current study by comparing prefrontal activation not only against a healthy control group, but also against clinical control groups (e.g. patients with depression).

## Supporting information

Höper et al. supplementary material Höper et al. supplementary material

## Data Availability

The data that support the findings of this study are available from the corresponding author, J.K., on reasonable request.
