# Supplementary material for: Prefrontal cortex activation under stress as a function of borderline personality disorder in female adolescents engaging in non-suicidal self-injury
Source: BJPsych Open. 2024 Aug 8;10(5):e142. doi: 10.1192/bjo.2024.728 (PMC11698208; doi:10.1192/bjo.2024.728)
Supplement: Höper et al. supplementary material [file S2056472424007282sup001.doc]

**Supplemental Material**

**Supplemental Table 1** Reasons for study exclusion

|  | NSSI group | HC group |
| --- | --- | --- |
| Age (18 and older) | 16 | 0 |
| Missing study informed consent | 16 | 0 |
| Acute substance use disorder | 16 | 0 |
| Male sex | 15 | 0 |
| BMI lower 17.5 or greater 30 | 9 | 2 |
| Study withdrawal | 9 | 8 |
| Diagnostic assessment older than 3 months | 8 | 0 |
| Medical treatment for BPD | 7 | 0 |
| Acute psychotic disorder | 3 | 0 |
| Neuroendocrinological diseases | 3 | 2 |
| Not showing up for appointment | 3 | 1 |
| Unsuccessful contact attempts | 2 | 3 |
| Acute suicidality | 1 | 0 |
| Inpatient treatment | 1 | 0 |
| Glucocorticoid drug intake | 1 | 2 |
| Pregnancy | 1 | 0 |
| Neurological diseases | 1 | 0 |
| Participation in former similar studies | 0 | 2 |
| Antihistamine intake | 0 | 1 |
| Further reasons | 3 | 0 |
| NSSI only: Less than five events of NSSI | 28 | - |
| HC only: Current or former psychiatric treatment | - | 5 |
| HC only: Lifetime events of NSSI or suicidality | - | 4 |

Note Supplemental Table 1. NSSI = non-suicidal self-injury; HC = healthy control; BMI = body mass index; BPD = borderline personality disorder.

**Supplement 1: Results from Exploratory Connectivity Analyses**

Cross-correlation coefficients for each time block (CDT, preparation TSST, free speech TSST, arithmetic task TSST) were calculated between all channels for each group. Results on connectivity are comprehensively summarized in Error: Reference source not found*.*

Supplemental Figure 1: Significant mixed models for connectivity analyses on O2Hb and with Borderline Personality Disorder dimensionality


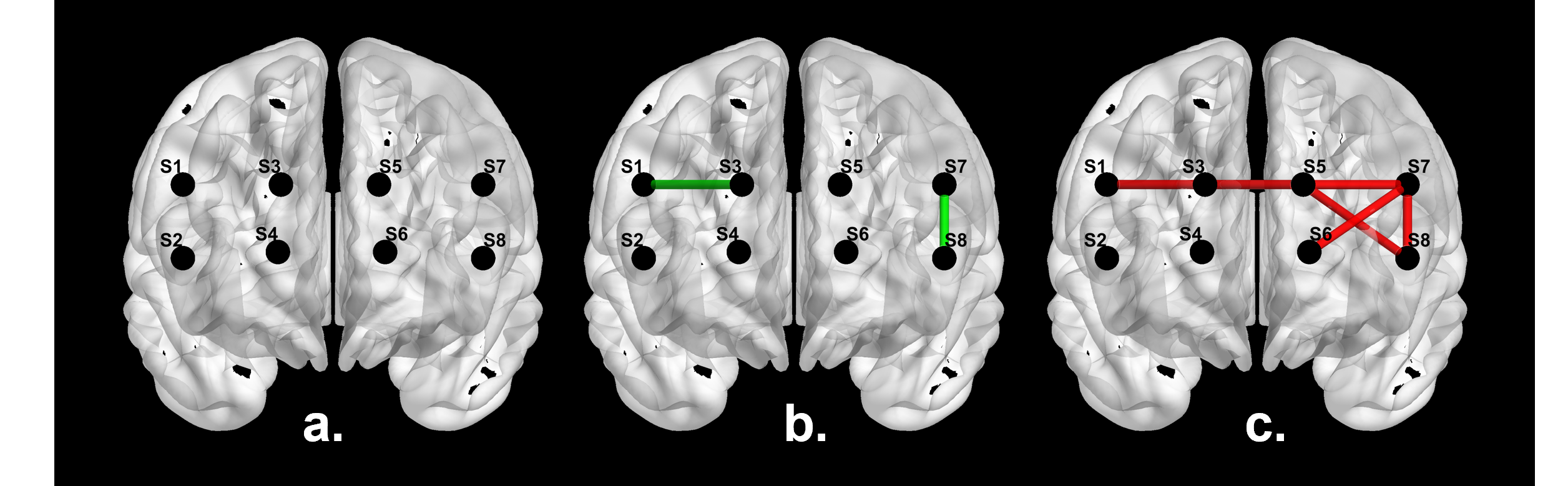


Note Supplemental Figure 1. a. – mixed model on O2Hb; b. – mixed model on O2Hb with the number of Borderline Personality Disorder criteria as predictor; c. – mixed model on O2Hb with the score of the Borderline Symptom Checklist (BSL-23) Score as predictor; displayed are p-values < .05.

These cross-correlation coefficients were included in a linear mixed-effects model for each channel pair with time block and group as predictors. None of the models reaches statistical significance (see Error: Reference source not found*,* brain template *a.*). Continuous models on BPD severity operationalized by the BSL-23 (see Error: Reference source not found, brain template *b.*) showed significant model fit for the connectivity of channel #S1 to channel #S3 with a significant TIME by SEVERITY interaction (Wald *χ^2^*(7) = 14.57; *p* = .042; BSL-23: *χ^2^*(3) = 9.89; *p* = .020), as well as for channel #S7 to #S8 but with no significant TIME by SEVERITY interaction (Wald *χ^2^*(7) = 17.28; *p* = .016; BSL-23: *χ^2^*(3) = 1.02; *p* = .795). In addition, continuous models on BPD severity operationalized by the number of BPD criteria (SCID-II; see Error: Reference source not found, brain template *c.*) showed significant model fit predicting the connectivity of channel #S1 to channel #S5 with no significant TIME by SEVERITY interaction (Wald *χ^2^*(7) = 14.26; *p* = .047; number of BPD criteria: *χ^2^*(3) = 6.82; *p* = .078). Furthermore, channel #S5 showed a significant model fit to channel #S7 with a significant TIME by SEVERITY interaction (Wald *χ^2^*(7) = 15.61; *p* = .029; number of BPD criteria: *χ^2^*(3) = 11.07; *p* = .011), to #S8 with no significant TIME by SEVERITY interaction (Wald *χ^2^*(7) = 14.77; *p* = .039; number of BPD criteria: *χ^2^*(3) = 4.91; *p* = .178), and from channel #S7 to #S6 with a significant TIME by SEVERITY interaction (Wald *χ^2^*(7) = 17.10; *p* = .017; number of BPD criteria: *χ^2^*(3) = 11.95; *p* = .008), and to #S8 with no significant TIME by SEVERITY interaction (Wald *χ^2^*(7) = 16.75; *p* = .019; number of BPD criteria: *χ^2^*(3) = 1.14; *p* = .767).
